# Reliable Route Selection for Wireless Sensor Networks with Connection Failure Uncertainties

**DOI:** 10.3390/s21217254

**Published:** 2021-10-31

**Authors:** Jianhua Lyu, Yiran Ren, Zeeshan Abbas, Baili Zhang

**Affiliations:** 1 School of Computer Science and Engineering, Southeast University, Nanjing 210096, China; 220201951@seu.edu.cn (Y.R.); ZeeshanAbbas5@hotmail.com (Z.A.); zhangbl@seu.edu.cn (B.Z.); 2 Key Laboratory of Computer Network and Information Integration (Southeast University), Ministry of Education, Nanjing 210096, China

**Keywords:** wireless sensor networks, connection failure uncertainty, route selection, reliable spanning tree, filtering

## Abstract

For wireless sensor networks (WSN) with connection failure uncertainties, traditional minimum spanning trees are no longer a feasible option for selecting routes. Reliability should come first before cost since no one wants a network that cannot work most of the time. First, reliable route selection for WSNs with connection failure uncertainties is formulated by considering the top-k most reliable spanning trees (RST) from graphs with structural uncertainties. The reliable spanning trees are defined as a set of spanning trees with top reliabilities and limited tree weights based on the possible world model. Second, two tree-filtering algorithms are proposed: the k minimum spanning tree (KMST) based tree-filtering algorithm and the depth-first search (DFS) based tree-filtering algorithm. Tree-filtering strategy filters the candidate RSTs generated by tree enumeration with explicit weight thresholds and implicit reliability thresholds. Third, an innovative edge-filtering method is presented in which edge combinations that act as upper bounds for RST reliabilities are utilized to filter the RST candidates and to prune search spaces. Optimization strategies are also proposed for improving pruning capabilities further and for enhancing computations. Extensive experiments are conducted to show the effectiveness and efficiency of the proposed algorithms.

## 1. Introduction

Wireless sensor networks (WSN) are widely used in various applications, such as military battlefield monitoring, traffic surveillance, environmental monitoring, intelligent agriculture, industrial system health management and transportation [1,2,3,4]. They are often deployed in cites that are not friendly to both human and chips. Moreover, the sensor nodes have limited resources on power, communication, computation and storage. Therefore, the communication channel between sensor nodes is susceptible to environmental events and node failures due to weather conditions, atmospheric qualities, moving obstacles and fabrication problems. Such connection failure obtains automatic recovery for the time being while the environment becomes normal. We regard them as **Wireless Sensor Networks with Connection Failure Uncertainties**. It is of great significance to develop route selection strategies for such WSNs in order to obtain all sensors connected with low cost and high reliability. We anticipate that a network with the highest reliability can serve for a longer period of time than the others possibly can.

Route selection for wired communication networks have been studied for decades and has become mature [5]. Connectivity and cost are two common objectives. Minimal spanning trees (MST) has been widely used to achieve minimal communication cost when all nodes are connected. However, cost is no longer the first consideration in addition to connectivity for WSNs with connection failure uncertainties. The route with minimal cost may serve only for a very short period of time since the connection edges it involves have relatively low existence probabilities. Consequently, reliability should come first before cost since no one wants a network that cannot work most of the time. A comprehensive objective for route selection is to find a route plan that is most reliable while its connection cost is upper-bounded by a practically accepted threshold. Actually, backup routes are also essential in cases where the most reliable route encounters a connection failure and can no longer be functional, which could be highly possible since every connection could fail with a certain probability.

There are many investigations devoted to route selection for WSNs [6,7,8,9,10,11,12,13,14,15,16]. Most of them assume that there are sink nodes that can transfer data with one another without any obstructions. Their main concern is to find suitable cluster heads as sink nodes. As a matter of fact, the inter-sink communications may also be unstable due to various disturbances in real applications. Reliable routing mechanisms for WSNs were also studied [17,18,19,20,21,22,23]. Reliability in these investigations was defined in terms of package loss ratio and transmission failure due to energy issues. Route selection on Zigbee networks was studied recently. Srikar and Benedito [24] focused on one-to-one and one-to-many reliable links. Kim and Chung [25] proposed a route management scheme for mobile networks to minimize control message collisions and delay time. All these works did not consider connection failure uncertainties.

Motivated by route selection methods for wired networks, we propose (1) modeling WSNs with connection failure uncertainties as uncertain graphs; and (2) searching reliable spanning trees (RST) from uncertain graphs as reliable communication routes. Uncertain graphs have structural uncertainties, and every weighted edge has a probability of existence, i.e., edge reliability. We propose using the possible world model [26] to define a reliable spanning tree. According to the possible world model, every spanning tree is a reliable spanning tree with a probability of existence. However, methods involving enumerating all spanning trees and testing their reliabilities and costs are not pragmatically acceptable. Therefore, the search space has to be reduced. After revisiting the MST searching algorithms [27,28,29], two MST-based tree-filtering algorithms and an efficient edge-filtering algorithm are proposed in order to improve RST’s searching efficiency from different angles.

To the best of our knowledge, we are the first to systematically investigate the route selection problem for WSNs with connection failure uncertainties.

The contribution of this paper is summarized as follows:

- The reliable route selection problem for WSNs with connection failure uncertainties is formulated by querying **top-k RSTs** from an uncertain graph, named **R-TopK Query**. RST reliability is defined based on the possible world model.
- Two tree-filtering algorithms for **R-TopK Query** processing are proposed: the k minimum spanning trees filtering algorithm and the depth-first search based tree-filtering algorithm, respectively. They are based on the traditional MST algorithms for deterministic graphs with new filtering and pruning techniques.
- An innovative edge-filtering **R-TopK Query** algorithm is proposed in which edge combinations that act as upper bounds for RST reliabilities are utilized to prune the search space. In order to improve pruning capabilities, several optimization strategies are introduced as well.
- Extensive experiments are conducted based on various datasets to show the effectiveness and efficiency of the proposed algorithms.

The rest of this paper is organized as follows. Section 2 introduces related investigations including existing WSN route selection methods, the possible world model and the MST finding algorithms on both deterministic and uncertain graphs. Section 3 presents some basic concepts, defines the spanning tree reliability and proposes the **top-k RSTs** problem to select reliable routes for WSNs with connection failure uncertainties. Two tree-filtering algorithms are discussed in Section 4. The edge-filtering algorithms and optimization techniques are explained in Section 5. Section 6 evaluates experimental results. Finally, conclusions and future works are discussed in Section 7.

## 2. Related Works

### 2.1. WSN Route Selection

There are many route selection methodologies for WSNs and Internet of Things (IoT). Clustering based data transmission model was proposed for energy optimization [7,8]. In order to address latency issues, fuzzy rules set for cluster head selection were discussed in [9,10]. The congestion controlling mechanism is studied in [11], with which the traffic load in WSNs could be mitigated. In [12], reliable data broadcasting for the IoT environment was proposed in order to obtain a secure routing mechanism and to cluster the network. A lightweight routing mechanism [13] was presented for routing Low-Power and Lossy Networks in order to obtain better energy efficiency and reduce delay and packet loss ratio. Buffer management and routing design were addressed for delay tolerant WSNs in [14,15,16]. They obtained better message replication, communication overhead and packet delivery performance. In [17,18,19], a mobile sink-based routing mechanism was proposed. An improved energy efficient LEACH-based routing approach for WSNs was proposed in [20], where cluster heads were selected based on node residual energy and average energy of WSNs. Most of these works investigated methods for selecting cluster heads in order to reduce communication costs.

Secure routing mechanisms for WSNs were also studied. A security trust-based model was proposed in [20], and a low latency energy efficient cluster-based multipath routing was presented in [21]. Multi-objective metrics for cluster head selection and multipath transmission for reliability and lifetime improvement were discussed in [22]. Furthermore, load balancing parameters were considered in routing metrics for providing reliability [23]. Route selection on Zigbee networks was studied by Srikar and Benedito [24], and they aimed to search for one-to-one and one-to-many reliable links. Kim and Chung [25] proposed a route management scheme for mobile networks in order to minimize control message collisions and delay time. In [30], a trade-off between improving lifetime performance and reliability requirement was discussed, and a reliable and energy efficient routing scheme for WSNs was proposed.

The reliability adopted in the existing studies were all defined in term of package loss ratio, transmission failure or delay time due to energy issues, which are different from the objectives of this paper.

### 2.2. Possible World Model

The possible world model was widely used for modeling uncertain data in various research fields [26,31,32]. The possible world space of an uncertain entity is composed of many possible world instances, which include deterministic values from uncertain entity attributes. A possible world instance is affiliated with a probability of the product of confidence of all attributes in the instance and the non-existing confidence of all attributes not in the instance. The sum of all instances’ probabilities should be one, which means that all possibilities are taken into account. For uncertain graph management, Yuan [33] and Zou [34] used a possible world model to study sub-graph searching and frequent sub-graph mining from uncertain graphs. The possible world semantic was also adopted by Yuan and Chen [35] for modeling the shortest paths over uncertain graphs. Liu, Jin and Aggarwal [36] studied the approaches for finding the most reliable clusters from uncertain graphs based on the possible world model.

### 2.3. Minimum Spanning Trees

There are many algorithms for finding minimum spanning trees from deterministic graphs [37,38]. The k minimum spanning tree (KSMT) problem was discussed in [27,28]. Enumerating all spanning trees in a deterministic graph was investigated in [39,40]. With uncertainties appearing in graphs, the traditional MST problem has to be revised [41,42,43]. The probabilistic minimum spanning tree (PMST) with vertex probabilities was proposed in [44]. Considering that the random variables are not enough for describing uncertainties, researchers studied the fuzzy minimum spanning tree (FMST) problem [45,46,47].

Zhang and Zou [29] discussed the most reliable minimum spanning tree problem on uncertain graphs. It is the most similar investigation with respect to the topics discussed in this paper. Considering that the brutal-force method needs to enumerate all possible world spanning trees and that time consumption grows exponentially with graph size, they proposed an approximate algorithm. Their contribution was finding one single minimum spanning tree and calculating its approximate probability efficiently. However, the main concern for WSN reliable route selection is searching several spanning trees with constrained costs and high probabilities.

## 3. Preliminaries and Problem Definition

WSNs with connection failure uncertainties are composed of several sensors and the sensors communicate with others via unstable channels. We first introduce an uncertain graph as the data model.

**Definition** **1** (Uncertain Graph). *An uncertain graph is a system G=(V,E,W,P), where V and E are the set of vertices and edges, respectively. Mapping W:E→R is a function assigning weights to edges, and P:E→(0,1] is a function assigning existence probabilities to edges.*

**Figure 1:**
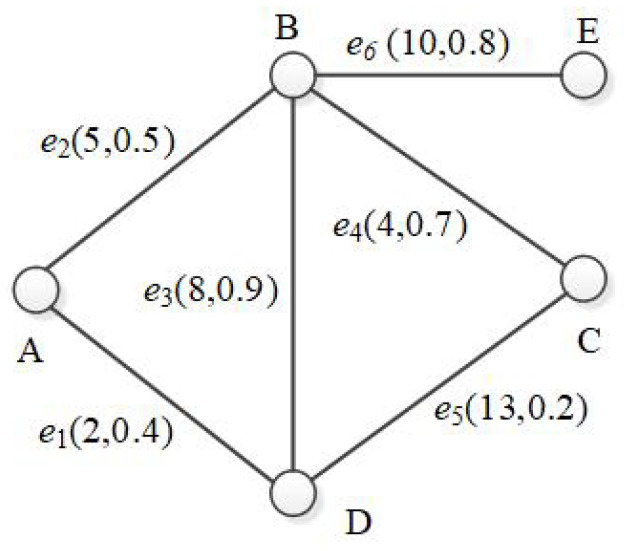
An example of uncertain graph: $G_{1}$.

Figure 1 shows an example of an uncertain graph. It models a WSN with five sensors and six communication channels. Sensor node **B** can connect to node **A**, **D**, **C** and **E**, costing 5, 8, 13 and 10, respectively. These connections are not stable, and each of them has a probability of existence. For example, vertex *B* and *E* cost 10 to be connected (W(<B,E>)=10) with a probability of 0.8, i.e. P(<B,E>)=0.8, which implies that the connection does not work 20% of the time.

By applying the possible world model, an uncertain graph implicates a set of deterministic graphs, and each one is a possible structure in which the uncertain graph may exist. Each possible structure is defined as a possible world graph.

**Definition** **2** (Derived Graph). *Given an uncertain graph G = (V, E, W, P), its derived graph G′ = (V, E, W, 1) is a deterministic graph with all edges existing, and it is denoted as $G\Rightarrow G^{\prime }$.*

For $G_{1}$ in Figure 1, its derived graph consists of all five vertices and all six edges with their existence possibilities all equal to 1.

**Definition** **3** (Possible World Graph). *Given an uncertain graph G = (V, E, W, P), a possible world graph $PW=(V,E^{\prime },W^{\prime })$ is a sub-graph of its derived graph G’, where $E^{\prime }\subseteq E$ and $W^{\prime }\subseteq W$. The relationship between G and PW is noted as G⇒PW, and the set composed of all the possible world graphs derived from G is denoted as PW(G).*

A possible world graph is a graph instance derived from an uncertain graph when the edges are deterministic.

For the sake of simplicity, we assume that all existence probabilities of edges are mutually independent. This assumption is reasonable in a range of practical applications [29,48,49,50]. Based on the independence assumption, the existence probability of a possible world graph PW implicated by *G* is defined as follows:

(1) $$P\left(G\Rightarrow PW\right)=\prod_{e\in E(PW)}P\left(e\right)\cdot \prod_{e^{\prime }\in E(G)-E(PW)}\left(1-P\left(e^{\prime }\right)\right).$$

where P(e) is the existence probability of any edge *e*.

**Figure 2:**
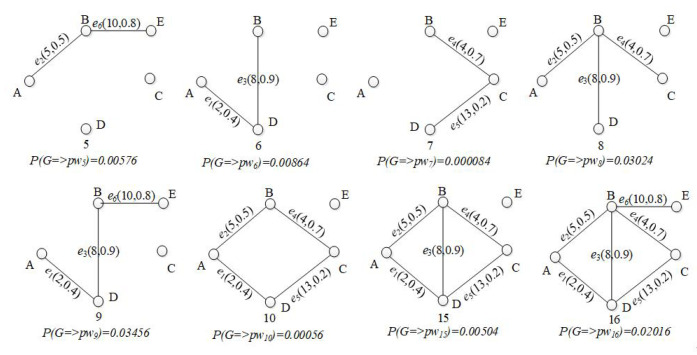
Some possible world graphs of $G_{1}$.

Figure 2 lists some of the possible world graphs derived from $G_{1}$ with their probabilities. For example, possible world graph $pw_{5}$ has two edges (<A,B>and<B,E>) and its probability of existence is 0.00576=0.5×0.8×(1−0.9)×(1−0.4)×(1−0.2)×(1−0.7).

The minimum spanning tree in a deterministic graph is defined as the one with the least amount of weight. In an uncertain graph, any spanning tree could be a minimum one since its cost is minimum at least in the possible world graph formed by itself. As a matter of fact, one spanning tree could be of minimum cost in multiple possible world graphs as long as (1) it is contained by all these possible world graphs and (2) its cost is the smallest among all the spanning trees of these possible world graphs. We define the reliable spanning tree (RST) and RST reliability based on all the possible world graphs it resides in.

**Definition** **4** (Reliable Spanning Tree, RST). *Given an uncertain graph G=(V,E,W,P), the corresponding possible world graph set PW(G) and a spanning tree T of $G\Rightarrow G^{\prime }$, where T is a Reliable Spanning Tree, and its reliability/probability are mathematically quantified as follows:*

(2) $$P_{RST}\left(T\right)=\sum_{PW\in PW(G)}P\left(G\Rightarrow PW\right)\cdot I_{1}\left(PW\right)\cdot I_{2}\left(PW\right).$$

*where $I_{1}\left(PW\right)$ and $I_{2}\left(PW\right)$ are indicator functions.*

$$I_{1}\left(PW\right)=\left\{\begin{array}{ccc} 1 & , & PW\;is\;connected,\\ 0 & , & otherwise. \end{array}\right.$$

$$I_{2}\left(PW\right)=\left\{\begin{array}{ccc} 1 & , & T\;is\;a\;MST\;of\;PW,\\ 0 & , & otherwise. \end{array}\right.$$

According to Definition 4, a RST could be the minimum spanning tree of multiple possible world graphs, and its reliability is the summation of these possible world graph probabilities. The computation cost of Equation (2) is high since the possible world graphs need to be enumerated and checked one by one.

In order to calculate RST reliabilities efficiently, we need to investigate RST in detail. For a minimum spanning tree, there are safe edges and dangerous edges [27]. We can similarly define RST safe edges and RST dangerous edges.

**Definition** **5** (Safe Edge and Dangerous Edge). *Given an uncertain graph G and $T\in RST(G\Rightarrow G^{\prime })$, for each e∈{E(G)−E(T)}, e is a safe edge if it has the largest weight in the cycle formed by adding e to T, and the safe edge set is noted as S(T); otherwise, e is a dangerous edge relative to T, and the dangerous edge set is noted as D(T).*

From Definition 5, we can observe that adding a dangerous edge into a possible world graph will render the original RST no longer reliable, while adding a safe edge will not induce the same impact. Thus, the uncertain minimum spanning tree theorem [29] is still valid for RSTs.

**Theorem** **1.** *Given an uncertain graph G=(V,E,W,P) and $T\in RST(G\Rightarrow G^{\prime })$, the probability of T being the minimum spanning tree of G can be calculated as follows.*

(3) $$P_{RST}\left(T\right)=\prod_{e\in E(T)}P\left(e\right)\cdot \prod_{e^{\prime }\in D\left(T\right)}\left(1-P\left(e^{\prime }\right)\right).$$

**Proof.**  The proof is quite similar to the one in reference [29].    □

Therefore, Equation (3) is equivalent to Equation (2). As a result, we can use Equation (3) to calculate RST reliabilities, which is more efficient for it finds edges that are not in a RST and checks their safety.

**Corollary** **1.** *Given an uncertain graph G=(V,E,W,P) and a reliable spanning tree T, the time complexity for calculating $P_{RST}\left(T\right)$ is O(|E||V|).*

**Proof.**  According to Theorem 1 and Definition 5, the procedure for calculating RST reliability includes three steps: (1) enumerating the edges; (2) checking if one edge is dangerous by cycle detection; and (3) combining the non-existence probability of the dangerous edges. Steps 1 and 3 can be conducted in one edge set scan, which is of time complexity O(|E|). The fast union-find algorithm [51] can be used to test whether one edge will cause a cycle or not, which is of worst time complexity O(|V|). Thus, the overall cost for calculating $P_{RST}\left(T\right)$ is O(|E||V|).    □

In order to facilitate WSN route selection, multiple routing strategies are needed in the case where there are connection failures in the serving route scheme. Accordingly, we must perform top-k reliable spanning tree query processing. There are many studies concentrated on top-k query processing [52,53]. We define **top-k RSTs** similarly as follows.

**Definition** **6** (Top-k RSTs). *Given an uncertain graph G, a positive integer* ***k*** *and a cost threshold ω∈R, the* ***top-k RSTs*** *of G are the first* ***k*** *spanning trees with top probabilities/reliabilities, and the tree weights are less than or equal to ω.*

*Suppose the reliability of the $k^{th}$ RST is λ, then we utilize **R-TopK Query**to find**top-k RSTs***.

(4) $$R-TopK\left(G,k,\omega \right)=\{T|W\left(T\right)\leq \omega ,P_{RST}\left(T\right)\geq \lambda ,T\in RST\left(G\Rightarrow G^{\prime }\right)\}.$$

**Figure 3:**
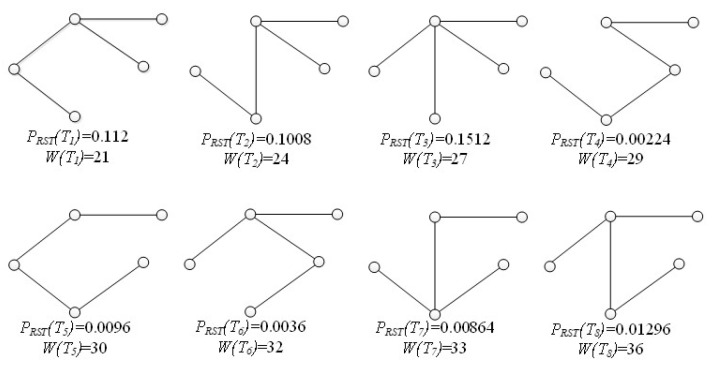
The possible RSTs of $G_{1}$.

Figure 3 lists all the candidate RSTs of $G_{1}$ with corresponding weights and reliabilities. Then, we obtain *R-TopK* ($G_{1},1,25)=\left\{T_{1}\right\}$ and *R-TopK* ($G_{1},2,30)=\left\{T_{3},T_{1}\right\}$. Even the backup route $T_{1}$ has a lower cost, and $T_{1}$ is the better choice for its higher reliability.

According to Definition 4 and Theorem 1, the brute force method of navigating all RSTs, calculating their reliabilities and then sorting is too time consuming. Thus, we propose reducing the search space by filtering out unqualified RSTs.

## 4. The Tree-Filtering Algorithms

In order to find the top-k RSTs, candidate RSTs are first generated, and then their reliabilities are tested for qualification.

### 4.1. The KMST Tree-Filtering Algorithm

**R-TopK Query** requires that tree costs/weights to be no higher than the given threshold ω. First, a candidate set composed of spanning trees with weights no higher than ω is generated. Then, the **top-k RSTs** are queried out by calculating and testing RST probabilities of the candidates.

First, we introduce the algorithm for finding the *k* minimum spanning trees in deterministic graphs [27]. The algorithm is called GENK, and it contains two procedures: EX and GEN. Suppose we have i−1 minimum spanning trees and we want to obtain the $i^{th}$ MST. First, EX finds a pair of edges [e,f], where $e\in {⋃}_{j=1}^{i-1}E\left(T_{j}\right)$, $f\in E\left(G\right)-{⋃}_{j=1}^{i-1}E\left(T_{j}\right)$ and W(f)−W(e) are minimums among all possible edge pairs. Then, GEN replaces *e* with *f* in order to obtain $T_{i+1}$. $T_{1}$ is the minimum spanning tree, and it is obtained by using Prim’s algorithm. $T_{i}$ can be obtained by exchanging edges based on the i−1 previously generated trees.

A straightforward and brute force method of obtaining the **top-k RSTs** is to seek the first *k* trees after sorting all the candidates on their probabilities. This is inefficient both in terms of space and time if the candidate set is large. In this paper, we use a priority queue, denoted as *Q*, to assist filtering and pruning procedures, e.g., reducing search space. *Q* is used to store the spanning tree candidates, and the size of *Q* is *k*. The priority in *Q* is the RST probability, and the spanning tree with top priority has the largest RST probability. The smallest probability in *Q* is used as the active filtering probability threshold ρ. As RSTs are inserted or removed from *Q* during query processing, the active filtering probability threshold may change. The filtering and pruning rules of the KMST tree-filtering algorithm(TF_KMST) are as follows:

- Rule 1: If size(Q)<k, call function GENK to generate the next spanning tree in weight ascending order and add it to *Q*.
- Rule 2: If size(Q)=k, call function GENK to generate the next spanning tree in weight ascending order. If the tree weight is not higher than ω, then calculate its RST probability and try to insert it into *Q*.
- Rule 3: The procedure stops when the weight of the newly generated spanning tree is higher than ω.

According to Rule 2, the newly generated spanning tree will not be inserted into *Q* when its probability is less than the active probability threshold. In TF_KMST, the search space contains all spanning trees, and the ones with weights higher than ω are pruned since function GENK generates spanning trees in weight ascending order, and the query procedure stops instantly by Rule 3 when the weight threshold is broken.

The pseudo codes of the KMST tree-filtering algorithm are outlined in Algorithm 1.
**Algorithm 1:** The KMST tree-filtering algorithm, TF_KMST**Input:** An uncertain graph G, an integer k, a weight threshold ω.**Output:** top-k RSTs.1: Q←⌀;ρ←0;T←Prim(G)2: **while** W(T)<ω **do**3:    T←GenNext()4:    ρ←Q.min().probability()5:    **if** Q.size()<k **then**6:        Q.insert(T)7:    **else if** P(T)>ρ **then**8:         Q.remove_min();Q.insert(T)9:    **end if**10: **end while**11: **return** Q.all()

As Algorithm 1 shows, TF_KMST enumerates all the spanning trees with weights no higher than ω. Time consumption grows linearly with the number of spanning trees generated, which is affected by weight threshold ω. TF_KMST enumerates all the spanning trees if ω is large enough. For a complete graph, the number of spanning trees is ${\left|V\right|}^{|V|-2}$, which is very large. However, if ω is small, the efficiency of TF_KMST will be very good. For each generated tree, we need to calculate its RST probability with time complexity O(|E||V|) according to Theorem 1.

### 4.2. The DFS Based Tree-Filtering Algorithm

The search space pruning rules of TF_KMST are based on the weight threshold ω. The cost is high when generating unqualified spanning trees and calculating their reliabilities. In order to avoid this, we consider utilizing tree reliabilities as pruning conditions in addition to the weight threshold. All the spanning trees of a deterministic graph can be enumerated based on the depth-first graph search strategy with time complexity O(|V|+|E|+N|V|) [40], where *N* is the number of spanning trees. By applying tree weights and tree reliabilities to the search space pruning when enumerating spanning trees, better performances may be achieved.

Algorithm ST_DFS lists all the spanning trees of a deterministic graph [40]. In ST_DFS, procedure GROW is used to find all the spanning trees that contain sub-tree Γ by using depth-first search. Initially, Γ only contains a root vertex *r*. By adding an edge $e_{1}$ incident to *r* to Γ in a depth-first manner, a new sub-tree $\Gamma =\Gamma \cup e_{1}$ with two vertices is produced. Then, a three-vertex sub-tree containing Γ by adding the second edge in a depth-first manner is obtained. Next, more sub-trees with more vertices are produced until a spanning tree is generated. By traversing the underlying graph in a depth-first manner, all the spanning trees are enumerated.

In ST_DFS, the spanning trees are generated by adding edges individually. Many sub-trees with fewer edges emerge first before obtaining an MST. Inspired by ST_DFS, we propose using sub-trees as upper bounds for the probabilities and as lower bounds for the RST weights for processing **R-TopK** queries. The pruning rules are as follows:

- Rule 1: If $W(\Gamma +e^{\prime })>\omega$, depth-first search starting from Γ via $e^{\prime }$ stops.
- Rule 2: If $\prod_{e\in E\left(\Gamma \right)\cup \left\{e^{\prime }\right\}}P\left(e\right)<\rho$ and the size of *Q* is *K*, depth-first search starting from Γ via $e^{\prime }$ stops.

(Correctness of Rule 1) According to Definition 6, the weights of the **top-k RSTs** must be smaller than ω. Any spanning tree obtained by using depth-first search starting from Γ via $e^{\prime }$ contains sub-tree $\Gamma +e^{\prime }$ with a weight larger than ω. Thus, the spanning trees containing $\Gamma +e^{\prime }$ are not qualified as the **top-k RSTs**. Rule 1 is correct for pruning further searches.

(Correctness of Rule 2) Similar to Rule 1, according to Theorem 1, the reliability of a spanning tree *T* generated from Γ via $e^{\prime }$ is as follows.

(5) $$P_{RST}\left(T\right)=\&\prod_{e\in E(T)}P\left(e\right)\cdot \prod_{e^{\prime }\in D\left(T\right)}\left(1-P\left(e^{\prime }\right)\right)\leq \prod_{e\in E(T)}P\left(e\right).$$

Since $E\left(\Gamma \right)\cup \left\{e^{\prime }\right\}\subseteq E\left(T\right)$ and 0≤P(e)≤1(e∈E(T)), we have $\prod_{e\in E(T)}P\left(e\right)\leq \prod_{e^{\prime }\in E\left(\Gamma \right)\cup \left\{e^{\prime }\right\}}P\left(e\right)<\rho$. Therefore, *T* is not qualified as one of the **top-k RSTs**. Rule 2 is correct.

**Figure 4:**
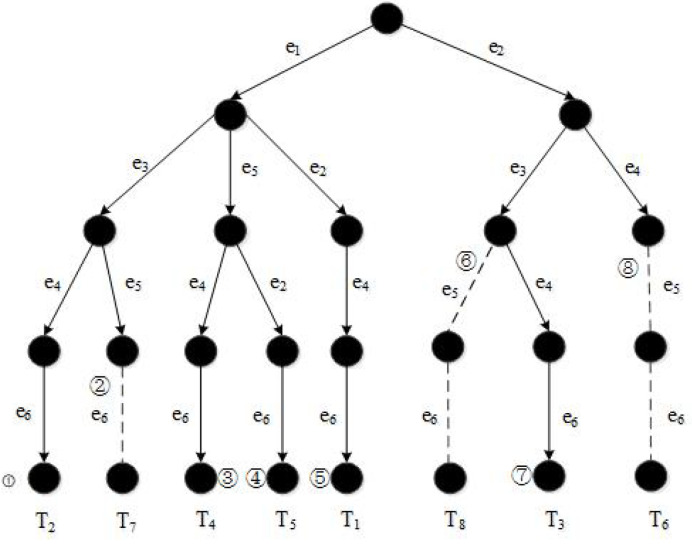
The pruning procedures in TF_DFS.

Figure 4 illustrates the pruning procedures of $G_{1}$ given in Figure 1. Here, k=2 and ω=30.

In Figure 4, the solid lines represent the recursive procedures of a depth-first search. The dashed lines are the procedures being pruned. $T_{2}$, $T_{4}$, $T_{5}$, $T_{1}$ and $T_{3}$ are generated and regarded as candidates (branch➀➂➃➄➆), while the procedures of generating $T_{7}$, $T_{8}$ and $T_{6}$(branches ➁➅➇) stop midway at certain points as pruning rules are applied.

Algorithm 2 provides the pseudo codes for the DFS based tree-filtering algorithm (TF_DFS).

ST_DFS takes time O(|V|+|E|+N|V|) and space O(|V|+|E|), where *N* is the number of spanning trees of *G*. TF_DFS may not list *N* trees because it can prune the search space by using the proposed two rules. We define $N^{\prime }\left(N^{\prime }\leq N\right)$ in TF_DFS to replace *N*. The RST probability is calculated for each of the listed spanning tree. In the worst cases, we need to adjust the priority queue $N^{\prime }$ times. The total time complexity of TF_DFS is $O(|V|+|E|+N^{\prime }|V|+N^{\prime }|E||V|+N^{\prime }\log k)$. $N^{\prime }$ is affected by parameter k and ω. The smaller k and ω are, the smaller $N^{\prime }$ is. When *k* is large enough, the second rule of TF_DFS may prune nothing.
**Algorithm 2:** The DFS based tree-filtering algorithm, TF_DFS**Input:** An uncertain graph G, an integer k, a weight threshold ω.**Output:** top-k RSTs.1: $Q\leftarrow ⌀;\rho \leftarrow 0;T\leftarrow \{V_{r}\}$2: **while** T←SD_DFS_GROW(T) **do**3:    ρ←Q.min().probability()4:    **if** W(T)>ω **then**5:        prune_rule1()6:    **else if** Q.size()==*k* and *T* is not a spanning tree and P(T)<ρ **then**7:        prune_rule2()8:    **else if** Q.size()<k and *T* is a spanning tree **then**9:        Q.insert(T)10:    **else if** P(T)>ρ and Q.size()==*k* **then**11:        Q.remove_min();Q.insert(T)12:    **end if**13: **end while**14: **return** Q.all()

## 5. The Edge-Filtering Algorithms

The proposed tree-filtering algorithms need to enumerate (minimum) spanning (sub-)trees and calculate their reliabilities. Every spanning tree of graph G=(V,E) can be represented as a set of edges of size |V|−1. Enumerating edge sets of size |V|−1 is easier than enumerating trees. We present an innovative strategy for **R-TopK Query** processing in which edge sets are utilized to filter RST candidates and to prune the search space.

### 5.1. Edge Combinations and Edge Filtering

First, we provide some definitions and introduce the basic idea of edge filtering.

**Definition** **7** (k-Edge-Combination). *Given an uncertain graph G=(V,E,W,P), a k-Edge-Combination is a subset of E with k distinct edges.*

The number of k-edge-combinations in *G* is $C_{\left|E\right|}^{k}$. Since a spanning tree of *G* has |V|−1 edges, we only consider the combinations of k=|V|−1. Without further information specified, we call (|V|−1)-Edge-Combination as an edge combination or combination for short, and we denote it as *C*.

**Definition** **8** (Combination Probability). *Given an edge combination C of G, the probability of C is defined as follows.*

(6) $$P\left(C\right)=\prod_{e\in E(C)}P\left(e\right).$$

**Proposition** **1.** *Let C be an edge combination of G; if C forms a spanning tree $T_{C}$, then $P_{RST}\left(T_{C}\right)\leq P\left(C\right)$.*

**Proof.**  From Equation (3), $P_{RST}\left(T_{C}\right)=\prod_{e\in E(C)}P\left(e\right)\cdot \prod_{e^{\prime }\in D\left(C\right)}\left(1-P\left(e^{\prime }\right)\right)$, then $P_{RST}\left(T_{C}\right)=P\left(C\right)\cdot \prod_{e^{\prime }\in D\left(C\right)}\left(1-P\left(e^{\prime }\right)\right)$. For every $e^{\prime }\in D\left(C\right)$, $0<P(e^{\prime })\leq 1$, then $0\leq (1-P\left(e^{\prime }\right))<1$. Thus, we have $P_{RST}\left(T_{C}\right)\leq P\left(C\right)$.    □

Proposition 1 shows the edge combination probability serves as the upper bound for the corresponding spanning tree reliability. Therefore, **Edge Filtering** can find **top-k RSTs** in three phases: (1) enumerating all edge combinations; (2) sorting the combinations in probability ascending order; and (3) filtering RST candidates using Proposition 1 and a priority queue.

Before presenting the details of **Edge Filtering**, we introduce an auxiliary data structure first. Let G=(V,E,W,P) be an uncertain graph in which *V* is the vertex set, and *E* is the edge set. A (|V|−1)-Edge-Combination *C* of *G* is represented as a bit vector *X* that fulfills the following:

- |X|=|E|;
- For $\forall e_{i}\in E\left(G\right)\left(1\leq i\leq |E|\right)$, if $e_{i}\in E\left(C\right)$, then X[i−1]=1; otherwise, X[i−1]=0;
- X[i,…,j] is empty when i>j.

By utilizing bit vector *X*, the method of enumerating all edge combinations of G(V,E,W,P) is as follows:

1. Step 1. Initialization: X[0] to X[|V|−2] are set to ‘1s’, and X[|V|−1] to X[|E|−1] are set to ‘0s’;
2. Step 2. Repeatedly scan *X* from left to right. Find the first ‘10’ sequence at position *i* and exchange the two bits to ‘01’. Move all ‘1s’ in sub-vector X[0...i−1] to the leftmost end of *X*;
3. Step 3. Enumeration stops when no ‘10’ exists.

Step 2 implies that the combinations are enumerated individually, and each combination except the first one is generated based on the previous combination.

The number of combinations grows exponentially with graph size and graph density. Therefore, the basic edge filtering cost is high when enumerating and sorting combinations, which is not acceptable.

### 5.2. Edge Filtering on the Fly

It is inefficient to generate, store and sort all the combinations because the total number of combinations may be extremely large. Therefore, we propose generating combinations on the fly and conducting filtering/pruning procedures at the same time.

Given an uncertain graph G=(V,E,W,P), we introduce some modifications to naive edge filtering, which result in obtaining the edge filtering algorithm on the fly, abbreviated as EF_OTF.

First, we rearrange bit vector *X* and categorized edge combinations into different groups:

- **Vector Rearrangement Rule**: For X[i](0≤i≤|E|−1) and X[j](0≤j≤|E|−1), if $P\left(e_{i}\right)>P\left(e_{j}\right)$, then i<j;
- **Edge Combination Grouping Rule**: Suppose $C_{i}$ is a combination of group $G_{k}$, denoted as $C_{i}\tilde{\in }G_{k}$. If there is only one sub-vector of ‘1s’ in *X* to the left of the first ‘10’ sequence but not at the leftmost positions, then $C_{i}$ is the last combination of $G_{k}$. $C_{i+1}$ generated based on $C_{i}$, and it is the first combination of next group, $G_{k+1}$.

The edges in bit vector *X* are arranged in probability descending order. Due to **Edge Combination Grouping Rules**, combinations within a group are arranged in probability descending order, which is the basis of filtering and pruning. As the second column of Table 1 shows, the combinations of size six with four ‘1s’ and two ‘0s’ are partitioned into five groups.

**Proposition** **2.** *Let $G_{k}$ be the $k^{th}$ group of edge combinations of G. If $C_{i}\left(C_{j}\tilde{\in }G_{k}\right)$ is generated before $C_{j}$, denoted as i<j, then $P\left(C_{i}\right)>P\left(C_{j}\right)$.*

**Proof.**  First, we consider two consecutive combinations, $C_{i}$ and $C_{i+1}$. By applying the combination enumerating method to the rearranged bit vector *X*, $C_{i+1}$ is generated from $C_{i}$ by replacing an edge of larger probability with another edge with smaller probability (‘10’ to ‘01’). Thus, for $C_{i}\tilde{\in }G_{k}$ and $C_{i+1}\tilde{\in }G_{k}$, $P\left(C_{i}\right)>P\left(C_{i+1}\right)$. Since the relation ’>’ is transitive, for any $C_{i}$ and $C_{j}\left(i<j\right)$, relationship $P\left(C_{i}\right)>P\left(C_{j}\right)$ holds.    □

Distinct from tree-filtering algorithms, EF_OTF applies four filtering or pruning rules for each combination $C_{i}\tilde{\in }G_{k}$ in EF_OTF:

- Rule 1: If $P\left(C_{i}\right)<\rho ,\forall C\in \left\{C_{j}|C_{j}\tilde{\in }G_{k},j>i\right\}canbesafelypruned;$
- Rule 2: If $W(C_{i})>\omega$, $C_{i}$ can be safely filtered;
- Rule 3: If $C_{i}$ cannot form a tree, $C_{i}$ can be safely filtered;
- Rule 4: If $P_{RST}\left(T_{C_{i}}\right)<\rho$, $C_{i}$ can be safely filtered.

Combination probabilities computation is simple and involves multiplying edge reliabilities. Tree testing of combinations requires vertex adjacency knowledge, while RST reliability calculation is appropriate only when there are trees. Rule 1 is the only rule to prune a search space, and its correctness can be deduced from Proposition 2. Therefore, we apply these four rules in order. The presto codes of EF_OTF are outlined in Algorithm 3.

In EF_OTF, the combinations generated later in the same group have lower chances of qualification for their lower reliabilities. For each combination, once it fails the condition in line 4, the first combination of the next group is produced (line 15), which means that the rest of the combinations in the current group are pruned from processing. When no combination is pruned, the worst time cost is O(N(|E||V|+|V|+logk)), where *N* is the number of combinations that are at most $C_{\left|E\right|}^{|V|-1}$.

We provide a running example of EF_OTF in Table 1. $G_{1}$ in Figure 1 is used and k=2 and ω=30. As Table 1 shows, the combinations are divided into five groups, and the combinations in each group are in probability descending order. Seven combinations are pruned because their probabilities are less than the active probability threshold ρ.
**Algorithm 3:** The Edge Filtering Algorithm On The Fly, EF_OTF**Input:** An uncertain graph G, an integer k, a weight threshold ω.**Output:** top-k RSTs.1: Q←⌀;ρ←0;sort(E);C←X.Initialize()2: **while** Cisnotthelastcombination **do**3:    ρ←Q.min().probability()4:    **if** P(C)>ρ **then**5:        **if** W(C)<ω and isTree(C) **then**6:           $P_{MST}\left(T_{C}\right)\leftarrow caculate\_MST\_Probability\left(T_{C}\right)$7:           **if** Q.size()<k **then**8:               $Q.insert(T_{C})$9:           **else if** $P_{MST}>\rho$ **then**10:               Q.remove_min();$Q.insert(T_{C})$11:           **end if**12:        **end if**13:        C←nextcombination14:    **else**15:        C←firstcombinationofnextgroup16:    **end if**17: **end while**18: **return** Q.all()

### 5.3. Multilayer Grouping Based Edge Filtering

In EF_OTF, the combinations within a groups are arranged in combination probability descending order, which is essential for search space pruning. The **Edge Combination Grouping Rule** is based on the combination enumerating process, where a new group begins whenever a sub-vector of ‘1s’ is shifted to the leftmost end. Let us consider Table 1 as an example. Shifting ‘111’ of ‘011101’ from position 1 to position 0 creates a new group starting with ‘111001’ (row 6), where ‘011101’ is the result of ‘1-0’ exchange of the previous group’s last combination ‘011110’ (row 5).

Algorithm EF_OTF tests the first combination of every group in order to determine whether the remaining combinations are pruned or not. It is inefficient when the number of groups is large. Therefore, a multilayer grouping strategy is proposed for enhancing pruning capacity.

We introduce a coding scheme for edge combinations that is also used for identifying groups.

**Definition** **9** (Combination Encoding). *Given an uncertain graph G=(V,E,W,P), |E|=m, |V|=n, C is an edge combination, and X⇔C is the corresponding bit vector of C under reliability order relation S. C is coded as $G\left(C\right)=G_{1}^{i_{1}}G_{2}^{i_{2}}...G_{d}^{i_{d}}$, 0≤d≤n−1, which is obtained by scanning X from right to left. The details are as follows.*

- *Definition: For a bit vector X, $r\_rank_{c}^{p}\left(X\right)$ is the number of bit ‘c’ in sub vector X[p...m−1], and $r\_select_{c}^{p}\left(X\right)$ is the index of the $p^{th}$ ‘c’ in X;*
- *Initialization: $s_{1}=0$ and $s_{2}=m-n+1$;*
- *Main procedure: Scan X from right to left repeatedly. Whenever a ‘1’ is encountered at position p, set $s_{1}=r\_rank_{0}^{p}\left(X\right)-s_{1}$ and $s_{2}=s_{2}-s_{1}$. Generate $G_{j}^{s_{2}}$ as one piece of combination code, where $j=r\_rank_{1}^{p}\left(X\right)$. If $s_{2}$ is equal to 1 or 0, the scanning process terminates; otherwise, the scanning continues;*
- *Output: The final code of G(C) is the concatenation of all generated encoding pieces.*

**Figure 5:**
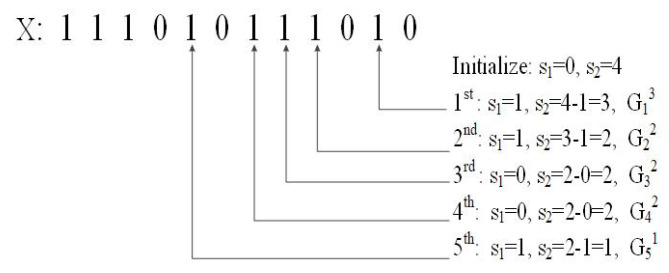
An example of combination encoding.

Figure 5 shows an example of edge combination encoding. For X⇔C, “111010111010”, m=12 and n=9, and the code of *C* is $G_{1}^{3}G_{2}^{2}G_{3}^{2}G_{4}^{2}G_{5}^{1}$.

**Definition** **10** (Multilayer Groups). *For $G\left(C_{i}\right)=G_{1}^{i_{1}}G_{2}^{i_{2}}...G_{d}^{i_{d}}$, there are d layers in which layer k is noted as $G_{k}^{i_{k}}$. Given $G(C_{i})$ and $G\left(C_{j}\right)=G_{1}^{j_{1}}G_{2}^{j_{2}}...G_{d}^{j_{d}}$, if $k(1\leq k\leq \min\left(d,d^{\prime }\right))$ exists such that $G_{1}^{i_{1}}G_{2}^{i_{2}}...G_{k}^{i_{k}}$ is equal to $G_{1}^{j_{1}}G_{2}^{j_{2}}...G_{k}^{j_{k}}$, then $C_{i}$ and $C_{j}$ are in the same group of layer k. In layer $k+1(1\leq k\leq \min\left(d-1,d^{\prime }-1\right))$, $C_{i}$ is in group $i^{k+1}$, and $C_{j}$ is in group $j^{k+1}$.*

**Proposition** **3.** *Given an edge combination C with code $G\left(C\right)=G_{1}^{l_{1}}G_{2}^{l_{2}}...G_{d}^{l_{d}}$ and the corresponding bit vector X, suppose $r\_rank_{0}^{p(i)}\left(X\right)-r\_rank_{0}^{p(i-1)}\left(X\right)=s_{i}$, where p(i) is $r\_select_{1}^{i}\left(X\right)$ and p(i−1) is $r\_select_{1}^{i-1}\left(X\right)$. Then, the maximum number of groups in layer k is as follows.*

(7) $$MaxGN\left(L_{k}\right)=\left\{\begin{array}{ccc} \; & m-n+1, & k=1,\\ \; & m-n+1-\sum_{j=1}^{k-1}s_{j}, & 1<k\leq d. \end{array}\right.$$

**Proof.**  From Definition 9 and Definition 10, we know that the layer index *k* in the multilayer grouping code of a combination is the number of ‘1s’ in X that have been scanned. The group index within layer *k* is the number of ‘0’s in *X* between the $k^{th}$ ‘1’ and the ${\left(k+1\right)}^{th}$ ‘1’ from right. Thus, when k=1, there are at most m−n+1 ‘0s’ not yet read, while there are at least $\sum_{j=1}^{k-1}s_{j}$ ‘0s’ that have been scanned when 1<k≤d. Thus, we have proven Equation (7). □

**Proposition** **4.** *Let SC be the set of all combinations of the given graph. $\forall C_{i},C_{j}\in SC\left(1\leq i<j\leq |SC|\right)$, if $C_{i}$ and $C_{j}$ are in the same group of layer k, and $C_{i}$ is generated before $C_{j}$, denoted as i<j, then we have $P\left(C_{i}\right)>P\left(C_{j}\right)$.*

**Proof.**  The proof is similar to that of Proposition 2. □

**Proposition** **5.** *Let $C_{i}$ and $C_{j}$ be two combinations, i<j, $G\left(C_{i}\right)=G_{1}^{l_{1}}G_{2}^{l_{2}}...G_{d}^{l_{d}}$, $l_{d}=0$, and $G_{1}^{l_{1}}G_{2}^{l_{2}}...G_{d-1}^{l_{d-1}}$ is a prefix of $G(C_{j})$; then, we have $P\left(C_{i}\right)>P\left(C_{j}\right)$.*

**Proof.**  From Definition 9, we know that when $l_{d}=0$, there is no ‘0’ between the ${\left(d-1\right)}^{th}$ ‘1’ and the $d^{th}$ ‘1’. Therefore, the first group of the $d^{th}$ layer contains only one combination $C_{i}$. Additionally, i<j means $C_{j}$ is generated after $C_{i}$, and $G(C_{j})$ has $G_{1}^{l_{1}}G_{2}^{l_{2}}...G_{d-1}^{l_{d-1}}$ but not $G_{1}^{l_{1}}G_{2}^{l_{2}}...G_{d-1}^{l_{d-1}}G_{d}^{0}$ as the prefix implies that there exists at least one more ‘0’ to the left of the ‘1’ corresponding to the $d^{th}$ piece of $G(C_{j})$ than that of $G(C_{i})$. Since the encoding procedure is from right to left and the edges in bit vector *X* are in probability descending order from left to right, we have $P\left(C_{i}\right)>P\left(C_{j}\right)$ according to Definition 8. □

According to Proposition 5, for group $G_{1}^{l_{1}}G_{2}^{l_{2}}...G_{d}^{l_{d}}$, there is only one combination when $l_{d}$ is equal to 0. We give the definition of succinct multilayer group identifier as follows. Without any ambiguities, we will use it to identify combinations and combination groups.

**Definition** **11** (Succinct Multilayer Combination/Group Encoding). *For $G\left(C\right)=G_{1}^{l_{1}}G_{2}^{l_{2}}...G_{d}^{l_{d}}$, if $l_{d}=0\left(d>1\right)$, G(C) is encoded as $G_{1}^{l_{1}}G_{2}^{l_{2}}...G_{d-1}^{l_{d-1}}$.*

**Proposition** **6.** *Given an edge combination C, $G\left(C\right)=G_{1}^{l_{1}}G_{2}^{l_{2}}...G_{d}^{l_{d}}$, there are four cases for locating the next group of G(C), noted as Next_G. The probability of the first combination of Next_G is not necessarily less than P(C).*

1. *If $l_{d}=1$ and d<n−1, Next_G is $G_{1}^{l_{1}}G_{2}^{l_{2}}...G_{d}^{l_{d}+1}G_{d+1}^{0}$;*
2. *If $l_{d}=1$, d=n−1 and $l_{d}+1\leq MaxGN\left(L_{d}\right)$, Next_G is $G_{1}^{l_{1}}G_{2}^{l_{2}}...G_{d}^{l_{d}+1}$.;*
3. *If $l_{d}=1$, d=n−1, $l_{k}\leq MaxGN\left(L_{k}\right)$ and $l_{k^{\prime }}>MaxGN\left(L_{k^{\prime }}\right)$ for all $k<k^{\prime }\leq d$, Next_G is $G_{1}^{l_{1}}G_{2}^{l_{2}}...G_{k}^{l_{k}+1}G_{k+1}^{0}$. If k=0, then Next_G receives nothing;*
4. *If $l_{d}=0$, Next_G is $G_{1}^{l_{1}}G_{2}^{l_{2}}...G_{d-1}^{l_{d-1}+1}G_{d}^{0}$. If $l_{k}\leq MaxGN\left(L_{k}\right)$ and $l_{k^{\prime }}>MaxGN\left(L_{k^{\prime }}\right)$ for all $k<k^{\prime }\leq d$, Next_G is $G_{1}^{l_{1}}G_{2}^{l_{2}}...G_{k}^{l_{k}+1}G_{k+1}^{0}$. If k=0, then Next_G is nil.*

**Proof.** (1): From Definition 9 and Definition 10, when $l_{d}=1$ and d<n−1, there is a ‘10’ changed to a ‘01’ from the last combination of the current group and the first combination of the next group. The bit ‘1’ is the basis of encoding $G_{d}^{?}$, so the next group is $G_{1}^{l_{1}}G_{2}^{l_{2}}...G_{d}^{l_{d}+1}G_{d+1}^{0}$. (2) and (3): When $l_{d}=1$ and d=n−1, every group in this layer has only one combination, so the next group is the next combination $G_{1}^{l_{1}}G_{2}^{l_{2}}...G_{d}^{l_{d}+1}$. Due to the fact that $l_{k}\left(k\leq d\right)$ exceeds the maximum capacity of layer, *k* implies that all of the groups of the current layer will run out, and we should consider the next bit ‘1’. Thus, we have to move to lower layers. (4) From Proposition 5, $l_{d}=0$ means the first combination of the next group is the first one not containing $G_{1}^{l_{1}}G_{2}^{l_{2}}...G_{d-1}^{l_{d-1}}$, so it should be $G_{1}^{l_{1}}G_{2}^{l_{2}}...G_{d-1}^{l_{d-1}+1}G_{d}^{0}$. The cases that $l_{k}\left(k\leq d\right)$ exceeds the maximum capabilities are similar to (3). □

Based on the combination encoding method, the multilayer grouping strategy and the multilayer group encoding based reliability upper bounding property introduced in Proposition 4 and Proposition 5, we propose the multilayer grouping-based edge filtering algorithm (EF_MLG) with the first pruning rule of EF_OTF replaced by the following pruning rule.

- **Multilayer Grouping based Pruning Rule**:
  If $P\left(C_{i}\right)<\rho ,\forall C\in \left\{C_{j}\right|i<k<j,G\left(C_{j}\right)\neq Next\_G$, $G(C_{k})=Next\_G\}$ can be safely filtered.

The algorithm structure of EF_MLG is similar to EF_OTF. We provide an example to show the pruning capability of EF_MLG as follows. Here, k=2 and ω=30. As Table 2 shows, there are four layers in the multilayer grouping of $G_{1}$ in Figure 1. The first layer contains three groups, and all the combinations belong to them. The third group of the first layer, denoted as $G_{1}^{2}$, is partitioned into three smaller sub-groups in the second layer. Sub-group $G_{1}^{2}G_{2}^{2}$ is further partitioned in the third layer and so on. As shown in row six of Table 2, when P(C)<ρ, the next combination group is located/generated based on Proposition 6. Since $G_{1}^{2}G_{2}^{0}$ is the first combination of group $G_{1}^{2}$ and does not fulfill the active reliability criterion, all the remaining combinations in this group are pruned. The example shows that EF_MLG has a better pruning capability than EF_OTF.

### 5.4. Optimization Strategies for Edge Filtering

The edge-filtering algorithms utilize edge combinations to act as an upper bound relative to tree reliabilities and creates more possibilities for optimizing.

#### 5.4.1. Optimized Priority Queue Initialization

The larger the initial active probability thresholds (ρ) in the edge-filtering algorithms, the better pruning capabilities they have. Both in EF_OTF and in EF_MLG, ρ is updated on the fly during combination enumerating and testing. They test RST candidates individually and may insert them into the priority queue, and the active ρ is updated. However, according to Proposition 2 and Proposition 4, combination probability decreases within groups, which results in the poor quality of the initial ρ obtained the first time, and the priority queue reaches its capacity *k*.

The combination grouping and multilayer grouping schemes allow us to locate all the first combinations from each group (in EF_OTF) or from each group in the first layer (in EF_MLG). These first combinations are in probability descending order according to the combination enumerating rules. Therefore, we use them to initialize the priority queue (i.e., through a top-k search over probabilities), which results in a bigger ρ at an early stage of the querying process introduced by the edge filtering algorithms.

#### 5.4.2. Bridge-Based Combination Space Reduction

In the edge-filtering algorithms, the number of edge combinations may be extremely large. Since each combination may need to be enumerated, tested and processed, it is important to develop combination space reduction strategies.

**Definition** **12** (Bridge). *Let G(V,E) be a connected graph for e∈E. e is a bridge if and only if graph G(V,E−{e}) and is not connected.*

Every spanning tree of a graph must contain all the bridge edges. Given graph *G* and its bridge edge set BE(G), the bridge-based combination space reduction rule is as follows.

- **Bridge Rule**: For all edges in BE(G), their corresponding bits in *X* are set to ‘1s’ during the combination enumerating procedure.

By applying the bridge rule, the total number of edge combinations is reduced to $C_{\left|E|-\right|E_{b}|}^{\left|V|-\right|E_{b}|}$.

New bridges may emerge after removing certain edges.

**Definition** **13** (Latent Bridge). *Given a connected graph G(V,E), e is a latent bridge covered by edge set Se if and only if Se is the smallest set of edges such that after removing them from graph G, e becomes a bridge edge noted as e=LB(Se).*

The latent bridge rule is as follows.

- **Latent Bridge Rule**: Given a latent bridge e=LB(Se), in all edge combinations with bits of Se reset to ‘0s’, the bit of e is set to ‘1’.

One latent bridge prunes away $C_{\left|E|-|Se\right|}^{\left|V|-|Se\right|}-C_{|E|-|Se|-1}^{|V|-|Se|-1}$ combinations. In practice, it is inefficient to find all latent bridges. In this paper, we only utilize latent bridges covered by single edges. For graph $G_{1}$, six combinations out of fifteen are pruned by the bridge-based space reduction rules.

#### 5.4.3. Cycle Indexing

In edge-filtering algorithms, edge combinations need to be tested when determining whether they are trees or not. An edge combination *C* forms a tree if it does not contain any cycles. Otherwise, it is not a tree.

**Figure 6:**
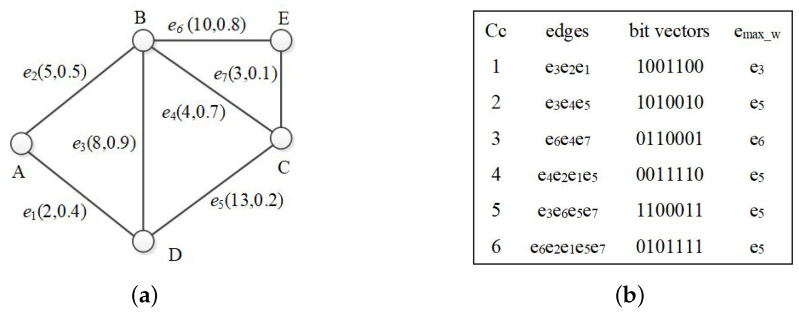
Uncertain graph and cycle information. (**a**) Uncertain graph $G_{2}$; (**b**) Cycle information of $G_{2}$.

We propose indexing cycles in order to improve the performance of combination tree testing. In order to be consistent and efficient, we use bit vectors to represent path cycles, the cycle bit vector is similar to the bit vector *X* used in EF_OTF and EF_MLG. A cycle Cc of *t* edges is of form t-Edge-Combination in which edges represented by bits are in reliability descending order. For example, cycle ’$e_{3}e_{2}e_{1}$’ is represented by a three-edge-combination bit vector ‘1001100’ as shown in Figure 6b.

**Definition** **14** (Edge Mask Code of Cycles). *Given a cycle Cc in G(V,E) and a set of edges Se⊆E, the mask code of Cc with respect to Se is a bit vector of length |E| by applying bitwise operator ‘AND’ (‘*&*’) on the bit vector of Cc and the |Se|-Edge-Combination of |Se|, noted as $MC_{Cc}\left(Se\right)$.*

According to Definition 14, if combination *C* contains one cycle Cc, for any edge subset Se of Cc, the result of ‘$C\&MC_{Cc}\left(Se\right)$’ contains at least one bit ‘1’.

For edge set $\left\{e_{1}\right\}$ of $G_{1}$, its bit vector is ‘0000100’. The mask code of cycle ’$e_{3}e_{2}e_{1}$’ with respect to $\left\{e_{1}\right\}$ is ‘0000100’ obtained by bitwise ANDing ‘1001100’ and ‘0000100’. Combination ‘1001101’ contains cycle “$e_{3}e_{2}e_{1}$”. The result of ANDing ‘1001101’ and mask code ‘0000100’ is ‘0000100’.

**(Cycle Indexing)** The bit vector-based cycle index structure, CCIndex_bv, is formed as follows:

- A CCIndex_bv is a four-level tree with three indexing levels of combination mask codes and one data level of edge combinations.
- The root of CCIndex_bv contains the mask codes from the rightmost ‘1’ of all cycles. Similarly, the nodes of the second and third levels contain the mask codes of second and third rightmost ‘1s’.
- The fourth level is the data level that stores all the cycle vectors.

**Figure 7:**
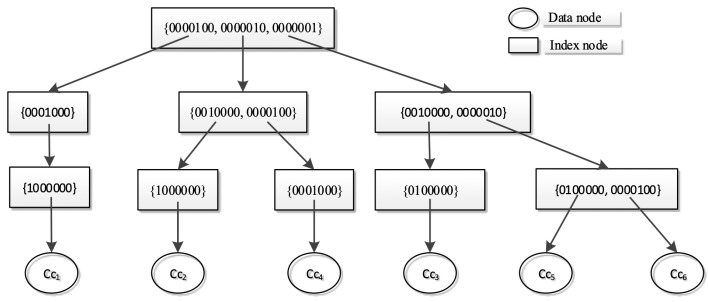
Index structure of cycles in $G_{2}$.

The bit vectors and the index structure of $G_{2}$ are shown in Figure 7.

The space overhead of CCIndex_bv is O(|E|+|CC|) regarding vectors, where |CC| is the number of cycles. The constructing times and worst searching times are O(|CC|). CCIndex_bv does not necessarily have three indexing levels, which can be up to |E|. The reason we chose three in this paper is that a cycle contains at least three edges.

The tree testing procedure of an edge combination *C* searches CCIndex_bv using bitwise operator ‘AND’ in a top-down manner. The search rules are as follows:

1. For each indexing node, test all the mask codes against *C*. If the result of operation ‘&’ is a vector containing bit ‘1’, then the corresponding subtree needs to be tested.
2. If no subtree needs to be tested, then *C* is a tree, and the tree testing terminates.
3. For each data node, if needed, test all the cycle vectors against *C*. If one result is equal to the cycle, then *C* is not a tree, and the tree testing terminates.

In the worst case, the entire tree is scanned. However, the search usually terminates midway.

When calculating a RST probability, each edge *e* needs to be tested as dangerous or not by (1) adding it to an RST to form a sub-graph $G_{s}$ with |V| edges (in the form of |V|-Edges Combination), (2) locating the cycles contained by $G_{s}$ and (3) testing *e* against the pre-calculated safe edges with maximum weights in cycles ($e_{max\_w}$ in Figure 6a). CCIndex_bv can facilitate steps (2) and (3).

## 6. Performance Evaluation

In this section, we present the experimental results for the effectiveness and efficiency of the algorithms we proposed.

### 6.1. Setups and Data Sets

(Platform settings) All algorithms were implemented by using the C++ programming language, with the help of the Standard Template Library (STL) and the Boost Library. The hardware was an Intel(R) Core(TM) i7-3770 @3.40 GHz CPU with 16GB main memory and MS Windows 7-x64.

(Datasets) The experiments were conducted on two datasets. One was a synthetic dataset generated by GraphGen [54], and the other was from a experimental WSN with Connection Failure Uncertainties named Ncfu. Ncfu had 29 cites and 60 connections, and each connection would fail according to a given reliability. Crossbow Imote sensors were used as network nodes since the communication channel can be programmed to avoid broadcasting. The connection costs were simulated by setting different transfer distances for the communication energy, and consumption is distance relevant. We used randomly moving blocks between connected sensors to simulate connection failures. The failure uncertainties were obtained by recording blocking frequencies.

(Parameters of weight) We used μ to set the weight threshold ω by $\omega =W\left(RST\right)+\mu *\bar{\omega }$, where W(RST) was the reliable spanning tree weight of *G*, and $\bar{\omega }$ was the average weight of all the edges in G. ω is linear to μ.

(Algorithms tested) There were six different algorithms tested including two tree-filtering algorithms, TF_KMST and TF_DFS, the naïve edge filtering algorithm EF_OTF, algorithm EF_Bridge that is the bridge-based combination space reduction version of EF_OTF, the multilayer grouping based edge filtering algorithm EF_MLG_Basic with bridge optimizing and the complete version of the multilayer grouping edge filtering algorithm EF_MLG with cycle indexing.

### 6.2. Performance Evaluation and Analysis

**Experiment 1.** Experiment 1 investigated the performance of the proposed algorithms on GraphGen with different graph sizes. This experiment was not conducted on Ncfu for its size was fixed.

Since the probabilities of edge combinations act as an upper bound to the corresponding RST reliabilities, many unqualified combinations were pruned by utilizing a priority queue in the edge filtering algorithms.

**Figure 8:**
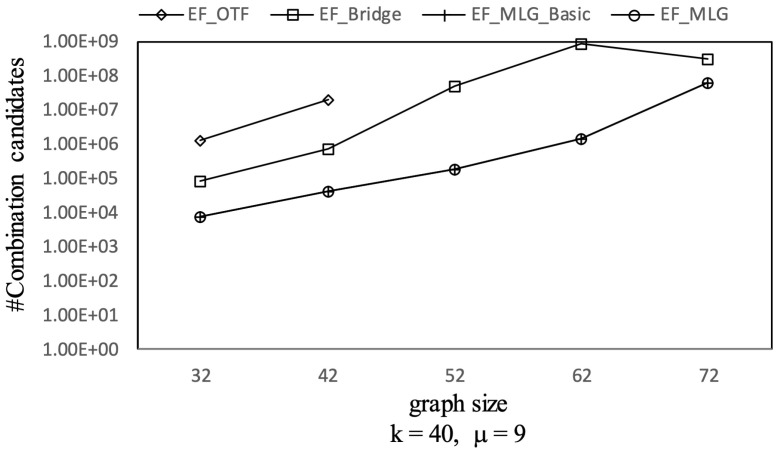
Combination pruning on GraphGen vs. graph size with *k* = 40 and μ = 9.

As Figure 8 shows, the combination pruning capabilities of EF_MLG and EF_MLG_Basic were the same since cycle indexing in EF_MLG was not proposed for pruning and were ten times better than EF_Bridge in terms of combination candidates by applying multilayer grouping on the datasets of different settings. Algorithm EF_Bridge outperformed EF_OTF by at least ten times in most cases since the bridge-based combination reduction rules were very effective. The total number of combinations increased exponentially as the graph size increased, while the increasing ratio of combination candidates was linear. The underlying reason is that the qualified combination candidates contain exactly |graphsize-1| edges, which is linear to graph size. The increasing trend did not hold in all cases. In certain cases, the significance of multilayer grouping was very large so that bigger graphs had fewer combination candidates. In most query settings, the number of combination candidates of EF_OTF was very large; thus, we did not show them for the sake of better readability.

**Figure 9:**
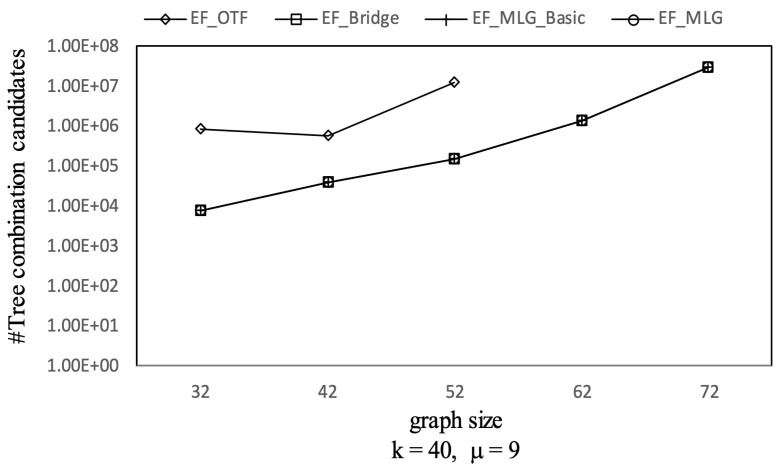
Tree combination pruning on GraphGen vs. graph size with with *k* = 40 and μ = 9.

Not every edge combination forms a tree. The edge filtering algorithms test the combination candidates first in order to determine whether they could form trees or not before compute the corresponding RST reliabilities. Figure 9 shows the tree combination pruning abilities of different edge filtering algorithms in terms of tree combination number. We can see EF_Bridge, EF_MLG_Basic and EF_MLG with the same query settings having the same #Tree-combination-candidates, which was at least 10 times smaller than that of EF_OTF. The underlying reason is the multilayer grouping in EF_MLG_Basic, and EF_ML is for combination pruning so that it can perform nothing for tree pruning, and the bridge-based combination reduction strategy adopted by the three algorithms except EF_OTF contributed to tree combination pruning all by itself and eliminated the un-tree combinations without bridge edges. We can also observe that the number of tree combination candidates increased when the graph size increased, except for the graph with 42 edges in which many of them were bridges. Similarly to Figure 8, we did not show some results of EF_OTF.

**Figure 10:**
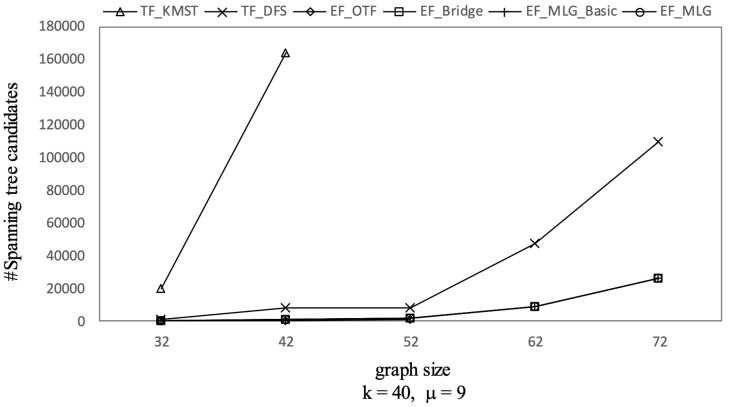
Spanning tree pruning on GraphGen vs. graph size with *k* = 40 and μ = 9.

Figure 10 shows the spanning tree pruning abilities of all the tree-filtering and edge-filtering algorithms in terms of spanning tree number. Algorithm TF_KMST was the worst for its pruning rule was only based on the weight threshold and did not consider tree probabilities that were crucial in **R-TopK** queries. Algorithm TF_DFS was much better since it utilized both the weights and probability thresholds to prune searching. The four edge filtering algorithms all had the same number of spanning-tree-candidates because the bridge-based, multilayer-grouping-based and indexing-based optimizations are all combination space pruning oriented. The advantages of EF_OTF, EF_Bridge, EF_MLG_Basic and EF_MLG are from the inherent essence of edge filtering that acts as upper bounds relative to RST reliabilities with combination probabilities.

**Figure 11:**
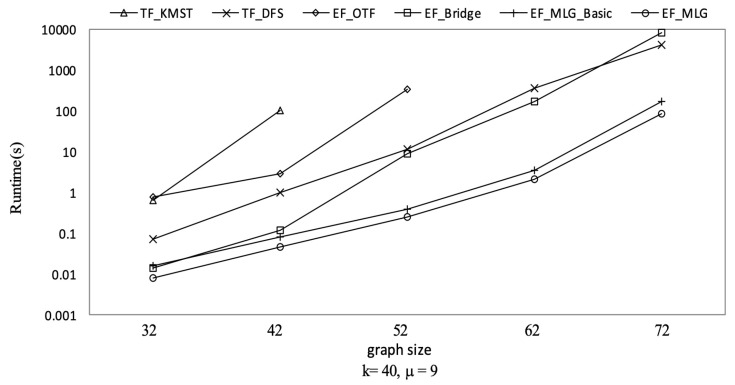
Runtime on GraphGen vs. graph size with *k* = 40 and μ = 9.

Figure 11 shows the running time of all proposed **R-TopK Query** processing algorithms. All costs increased exponentially when the graph size increased. The reason is that there were more combination candidates, more tree candidates and more computing tasks as the graph size increased.

TF_KMST was the worst algorithm. TF_DFS outperformed EF_OTF since there were too many EF_OTF combination candidates. When the graph sizes were small, the combination space was also small; EF_Bridge was better than TF_DFS. However, the superiority did not hold when there were few bridges, e.g., in dense graphs.

Multilayer grouping was proved to be efficient in Figure 11 by showing EF_MLG_Basic and EF_MLG were at least 10 times faster than the others. The bit vector-based cycle indexing made EF_MLG two to three times faster than EF_MLG_Basic.

**Experiment 2.** This experiment tested the performance on GraphGen and Ncfu when parameter *k* varied.

**Figure 12:**
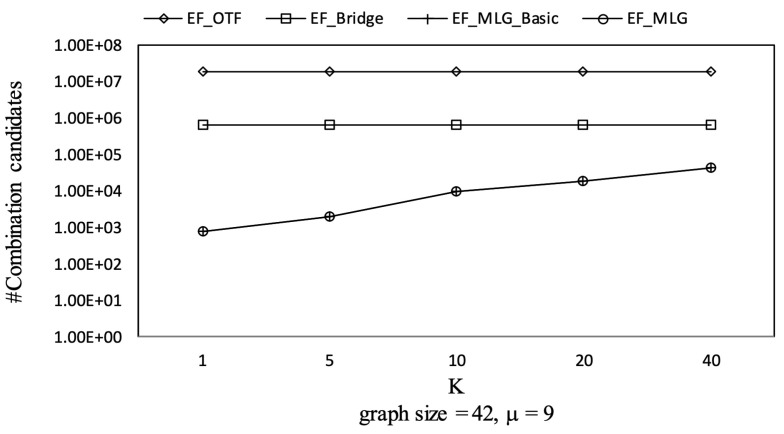
Combinations pruning on GraphGen vs. *k* with graphsize = 42 and μ = 9.

**Figure 13:**
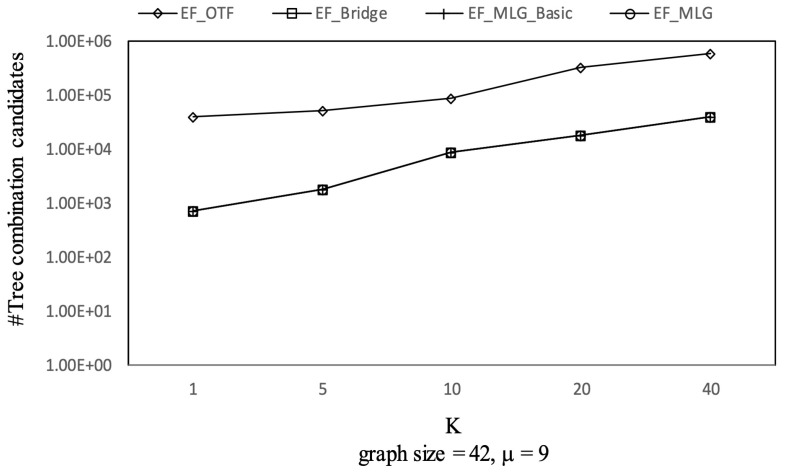
Tree combination pruning on GraphGen vs. *k* with graphsize = 42 and μ = 9.

**Figure 14:**
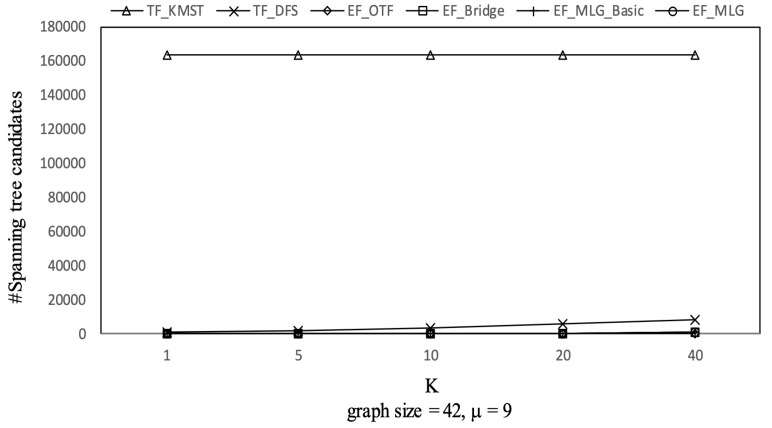
Spanning tree pruning on GraphGen vs. *k* with graphsize = 42 and μ = 9.

Figure 12, Figure 13 and Figure 14 show the pruning capabilities of the proposed algorithms with a different parameter *k*. EF_OTF and EF_Bridge had so many groups that the increase in ρ caused by a larger *k* had very little impact. EF_MLG_Basic and EF_MLG were affected more by *k* for multilayer grouping and had fewer groups. A larger *k* implies a bigger active ρ so that the inter-group jumping occurred more frequently in the lower layers, resulting in poor pruning effects. In Figure 12, EF_MLG_Basic and EF_MLG were in line for cycle indexing and cannot filter combinations. In Figure 13, EF_Bridge, EF_MLG_Basic and EF_MLG were in line since multilayer grouping can perform nothing on filtering tree combinations. In Figure 14, EF_OTF, EF_Bridge, EF_MLG_Basic and EF_MLG were in line because bridges were only used for filtering un-tree combinations.

**Figure 15:**
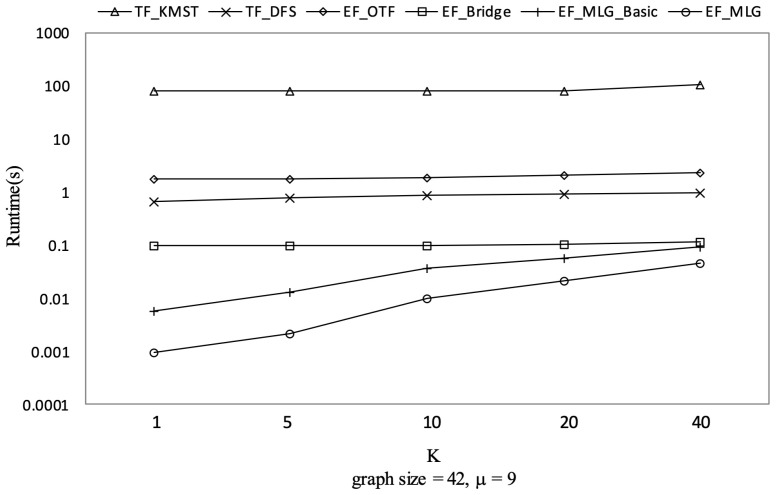
Runtime on GraphGen vs. *k* with graphsize = 42 and μ = 9.

TF_KMST had an almost identical number of spanning tree candidates as Figure 14 shows since *k* was not used in pruning. Therefore, we can observe its steady but poor performance in Figure 15. TF_DFS was also not affected by *k* since a larger k here only meant a larger active ρ but not a larger initial ρ which was much more important to the pruning procedure in TF_DFS.

**Figure 16:**
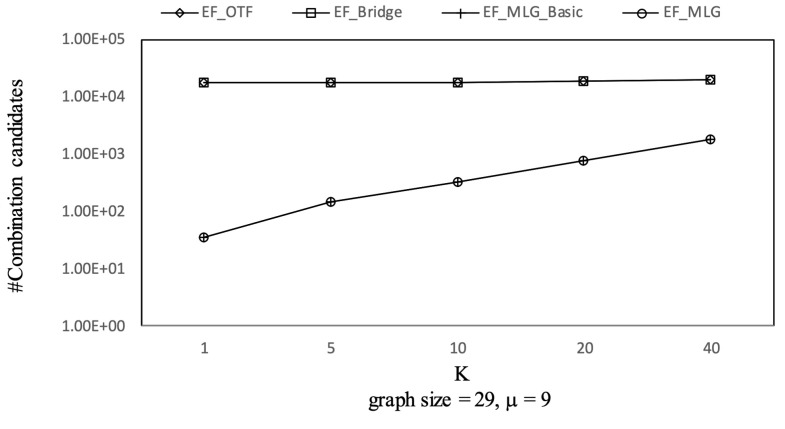
Combination pruning on Ncfu vs. *k* with graphsize = 29 and μ = 9.

**Figure 17:**
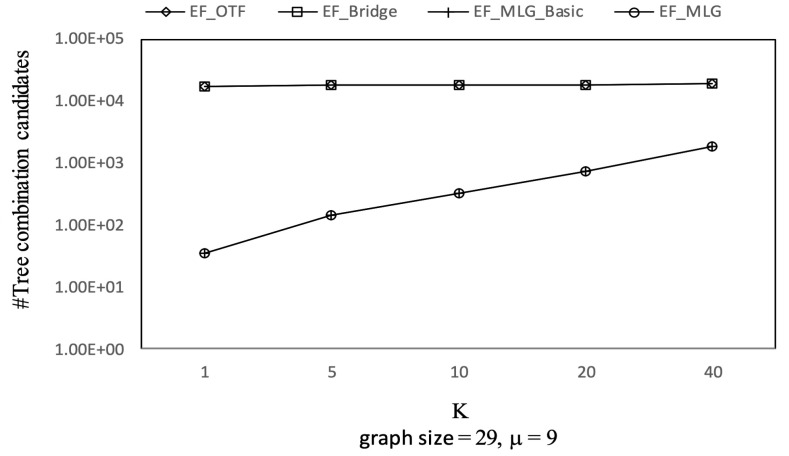
Tree combination pruning on Ncfu vs. *k* with graphsize = 29 and μ = 9.

**Figure 18:**
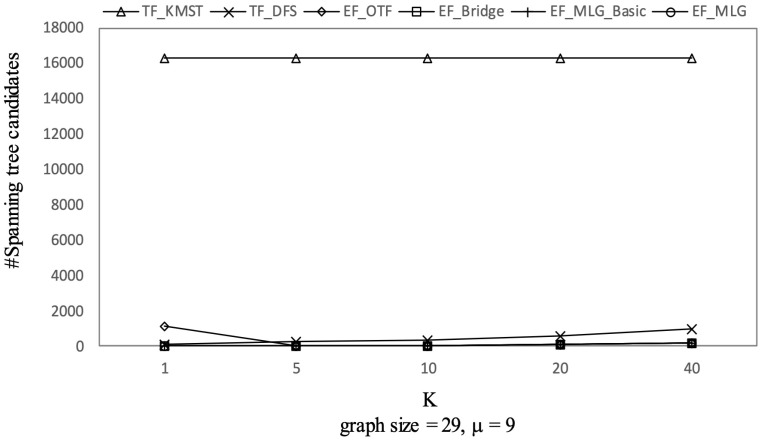
Spanning tree pruning on Ncfu vs. *k* with graphsize = 29 and μ = 9.

**Figure 19:**
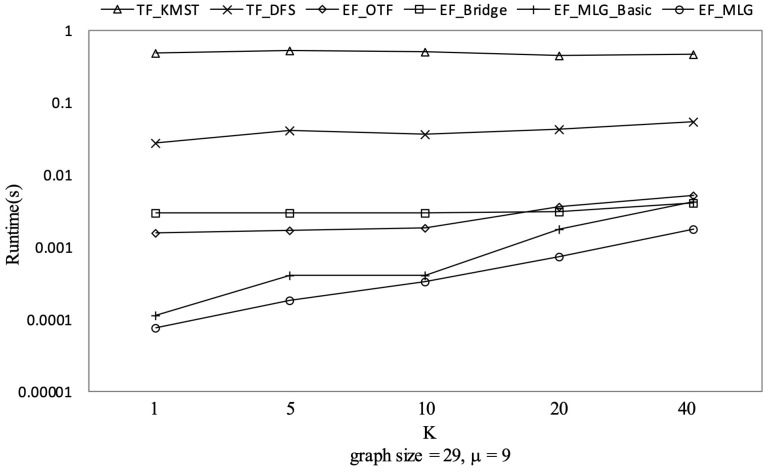
Runtime on Ncfu vs. *k* with graphsize = 29 and μ = 9.

The experimental results on Ncfu are shown in Figure 16, Figure 17, Figure 18 and Figure 19. The overall trends were similar to those from GraphGen. We noticed that there was no bridge in Ncfu; thus, the bridge-based pruning did not work in the edge filtering algorithms. Algorithm EF_MLG was the best algorithm, while its superiority relative to EF_MLG_Basic weakened. The reason is that, as a sparse graph, the ratio of cycles of Ncfu was lower than that of GraphGen. There are also many in-line curves, and the reasons are the same to that of experiments on GraphGen.

**Experiment 3.** Experiment 3 evaluated the performance on GraphGen and Ncfu when μ changed.

**Figure 20:**
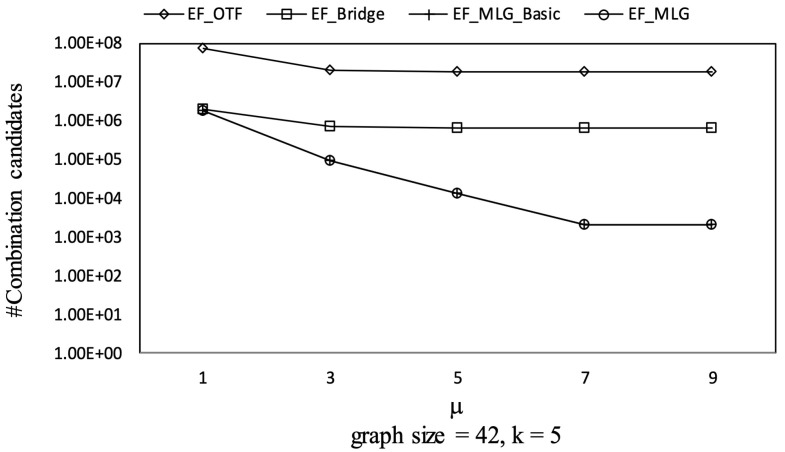
Combination pruning on GraphGen vs. μ with graphsize = 42 and *k* = 5.

As had been analyzed, a larger initial ρ resulted in better pruning capability. In the edge filtering algorithms with optimized priority queue initializing, the initial ρ was obtained by scanning the first combinations in all the groups. According to the introduced combination enumerating rules and the grouping rules, in addition to the local order within the groups, there is a global order among the first combinations that is used in multilayer grouping and the optimized priority queue initialization. Therefore, by applying larger weight thresholds, the first combinations with larger global orders were more involved in the determination of the initial ρ. Therefore, the combination pruning capability would be better. This is why the runtime of the edge filtering algorithms had decreasing trends as μ increased in Figure 20 and Figure 23.

**Figure 21:**
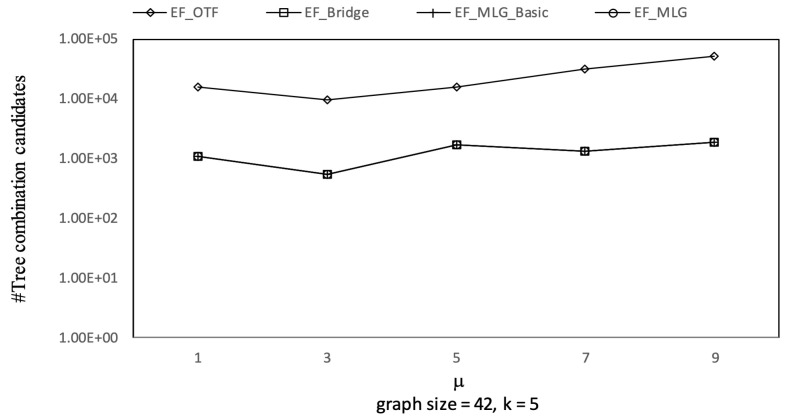
Tree combination pruningon GraphGen vs. μ with graphsize = 42 and *k* = 5.

**Figure 22:**
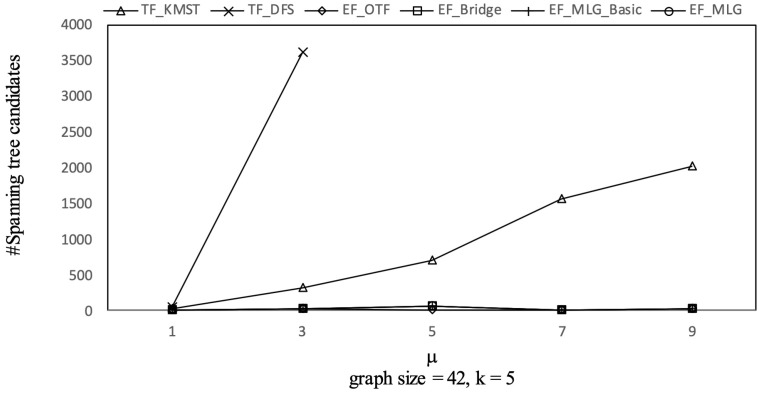
Spanning tree pruning on GraphGen vs. μ with graphsize = 42 and *k* = 5.

The overall trends of tree combination candidates shown in Figure 21 are increasing since there were more qualified tree combinations with a bigger μ. As long as there are *k* RSTs, μ does not affect the spanning tree candidates. Thus, in Figure 22, there are no clear deviations except for TF_KMST and TF_DFS.

**Figure 23:**
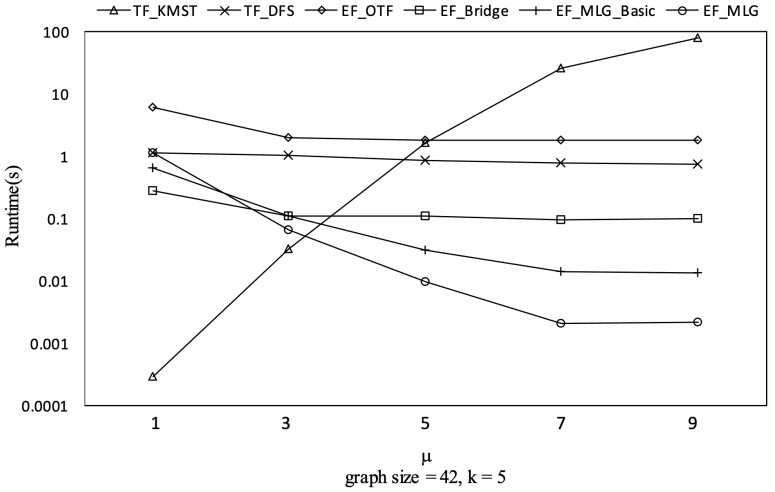
Runtime on GraphGen vs. μ with graphsize = 42 and *k* = 5.

However, if μ becomes large enough such that all the necessary first combinations for finding the best initial ρ are obtained, the number of combination candidates will no longer decrease. TF_KMST was the fastest with a small μ since it only needs to enumerate a few RST candidates as shown in Figure 23.

**Figure 24:**
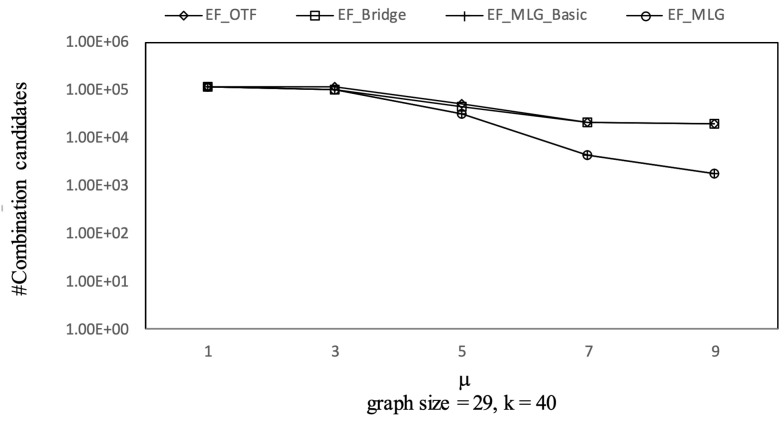
Combinations pruning on Ncfu vs. μ with graphsize = 29 and *k* = 40.

**Figure 25:**
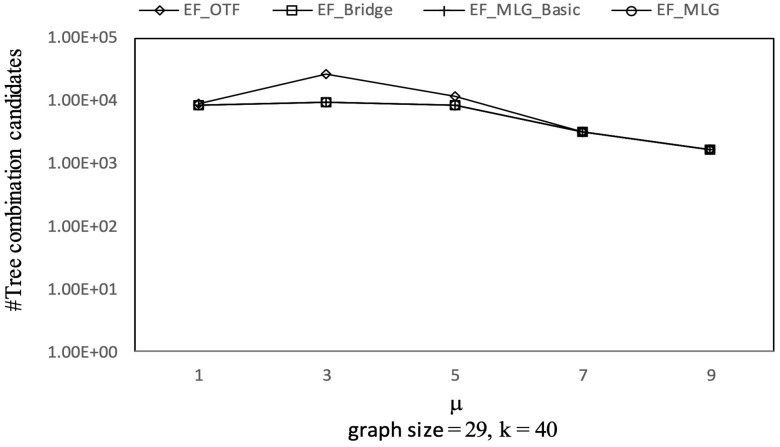
Tree combination pruning on Ncfu vs. μ with graphsize = 29 and *k* = 40.

**Figure 26:**
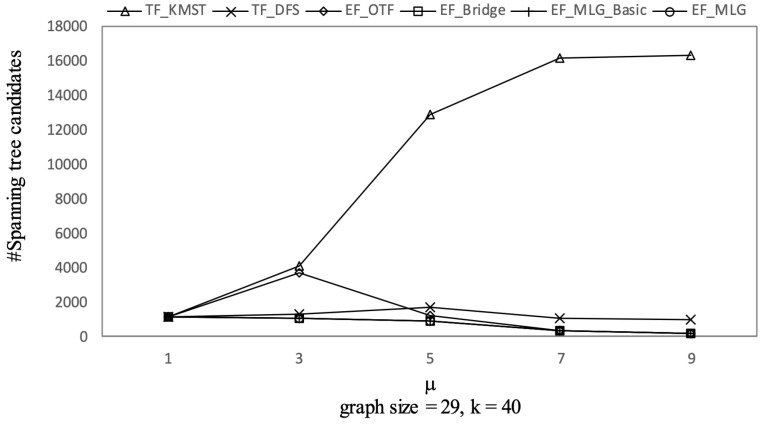
Spanning tree pruning on Ncfu vs. μ with graphsize = 29 and *k* = 40.

**Figure 27:**
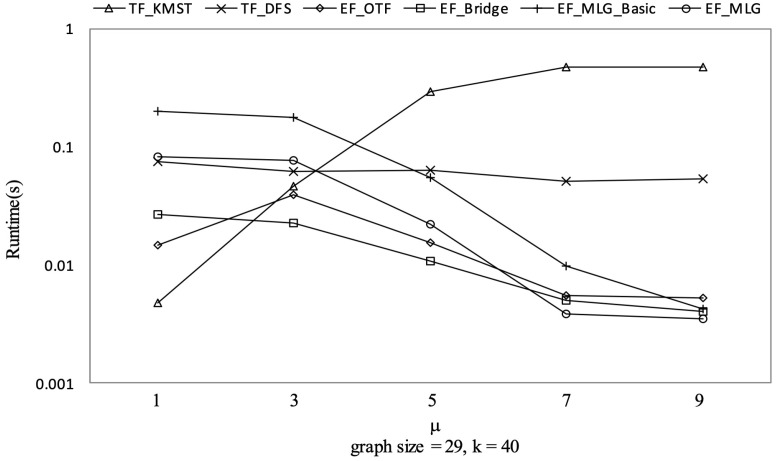
Runtime on Ncfu vs. μ with graphsize = 29 and *k* = 40.

Figure 24, Figure 25, Figure 26 and Figure 27 show the experimental results on Ncfu, in which there was one difference from the synthetic dataset. We can observe that TP_KMST costs slightly more when μ increased from 7 to 9. The reason is that for a small and sparse graph such as Ncfu, μ was larger than seven in TF_KMST, which means enumerating almost all the RSTs such that a different μ makes little difference.

There are also many in-line curves from Experiment 3, such as EF_MLG_Basic and EF_MLG have the same combination pruning abilities. EF_Bridge, EF_MLG_Basic and EF_MLG have the same tree combination pruning performance. EF_OTF, EF_Bridge, EF_MLG_Basic and EF_MLG are no different on spanning tree pruning. The reasons are studied when analyzing Experiments 1 and 2.

## 7. Conclusions and Future Works

This paper explores route selection for WSNs with connection failure uncertainties by searching the **top-k RSTs** in uncertain graphs. Two tree-filtering algorithms are proposed. TF_KMST is inefficient for only using tree weights to prune the search space, so TF_DFS utilizes both tree reliabilities and tree weights when traversing and testing. Its performance is acceptable on small graphs, but it is poor when the graph size increased. Edge combination is then proposed as its probability upper bounds RST reliability. It can identify a small set of spanning tree candidates but is still inefficient. Various performance optimization techniques including multilayer grouping, edge combination space reducing and cycle indexing are introduced. Extensive experiments on both synthetic and simulation datasets are conducted. The evaluation and analysis show the superiority of edge filtering relative to tree filtering in most of the cases. To conclude, TF_DFS is a performance steady method; TF_KMST is suitable for cost limited scenarios where the acceptable weight threshold is low; EF_MLG is recommended for most of the occasions; and a larger weight threshold is always preferred.

The environmental dynamics of WSNs may change over time. Then, the connection failure uncertainties may not be consistent. For example, in a WSN deployed for logistical field monitoring, the reliability of one connection could rise when there are fewer vehicles crossing between the two sensors. Therefore, one of the future works is to develop online update strategies for obtaining new **top-k RSTs** based on the out-of-date ones. Some WSNs may have multiple-state connection failure uncertainties, where a connection has more than one connecting cost, and each cost has a corresponding probability. For example, the sensors can have multiple communication channels or protocols. Then, the entire framework should be re-studied, including the uncertain graph model, definition of reliability and **top-k RSTs** query processing algorithms.

## Figures and Tables

**Table 1:** A running example of EF_OTF.

Group	X	C	ρ	P (C)	$P_{M}\mathrm{ST}\left(T_{C}\right)$	Description
$G_{1}$	111100	$e_{3}e_{6}e_{4}e_{2}$	0	0.252	0.1512	Update Q
	111010	$e_{3}e_{4}e_{6}e_{2}$	0.1512	0.2016	0.1008	Update Q
	110110	$e_{3}e_{6}e_{2}e_{1}$	0.1008	0.144	Not tree	Next C
	101110	$e_{3}e_{4}e_{2}e_{1}$	0.1008	0.126	Not tree	Next C
	011110	$e_{6}e_{4}e_{2}e_{1}$	0.1008	0.112	0.112	Update Q
$G_{2}$	111001	$e_{3}e_{6}e_{4}e_{5}$	0.112	0.1008	Not tree	Next Group
	110101	$e_{3}e_{6}e_{2}e_{5}$	−	0.072	−	Pruned
	101101	$e_{3}e_{4}e_{2}e_{5}$	−	0.063	−	Pruned
	011101	$e_{6}e_{4}e_{2}e_{5}$	−	0.056	−	Pruned
$G_{3}$	110011	$e_{3}e_{6}e_{1}e_{5}$	−	0.057	−	Next Group
	101011	$e_{3}e_{4}e_{1}e_{5}$	−	0.00504	−	Pruned
	011011	$e_{6}e_{4}e_{1}e_{5}$	−	0.0448	−	Pruned
$G_{4}$	100111	$e_{3}e_{2}e_{1}e_{5}$	0.112	0.036	−	Stop
	010111	$e_{6}e_{2}e_{1}e_{5}$	−	0.0032	−	Pruned
$G_{5}$	001111	$e_{4}e_{2}e_{1}e_{5}$	−	0.0028	−	Pruned

**Table 2:** A running example of EF_MLG.

Group	X	1st	2nd	3rd	4th	ρ	P (C)	$P_{\mathrm{MST}}\left(T_{C}\right)$	Description
$G_{1}^{0}$	111100	$G_{1}^{0}$				0	0.252	0.1512	Update Q
$G_{1}^{1}$	111010	$G_{1}^{1}$				0.1252	0.2016	0.1008	Update Q
$G_{1}^{1}$	110110					0.1008	0.144	Not tree	Next C
$G_{1}^{1}$	101110					0.1008	0.126	Not tree	Next C
$G_{1}^{1}$	011110					0.1008	0.112	0.112	Update Q
$G_{1}^{2}G_{2}^{0}$	111001	$G_{1}^{2}$	$G_{2}^{0}$			0.112	0.1008	Not tree	Next_G
$G_{1}^{2}G_{2}^{1}$	110101		$G_{2}^{1}$			-	0.072	-	Pruned
$G_{1}^{2}G_{2}^{1}$	101101					-	0.063	-	Pruned
$G_{1}^{2}G_{2}^{1}$	011101					-	0.056	-	Pruned
$G_{1}^{2}G_{2}^{2}G_{3}^{0}$	110011		$G_{2}^{2}$	$G_{3}^{0}$		-	0.0576	-	Pruned
$G_{1}^{2}G_{2}^{2}G_{3}^{1}$	101011			$G_{3}^{1}$		-	0,00504	-	Pruned
$G_{1}^{2}G_{2}^{2}G_{3}^{1}$	011011					-	0.0448	-	Pruned
$G_{1}^{2}G_{2}^{2}G_{3}^{2}G_{4}^{0}$	100111			$G_{3}^{2}$	$G_{4}^{0}$	-	0.036	-	Pruned
$G_{1}^{2}G_{2}^{2}G_{3}^{2}G_{4}^{1}$	010111				$G_{4}^{1}$	-	0.0032	-	Pruned
$G_{1}^{2}G_{2}^{2}G_{3}^{2}G_{4}^{2}$	001111				$G_{4}^{2}$	-	0.0028	-	Pruned
